# *Staphylococcus aureus* Sensitivity to Membrane Disrupting Antibacterials Is Increased under Microgravity

**DOI:** 10.3390/cells12141907

**Published:** 2023-07-21

**Authors:** Hyochan Jang, Seong Yeol Choi, Robert J. Mitchell

**Affiliations:** School of Life Sciences, Ulsan National Institute of Science and Technology, Ulsan 44919, Republic of Korea

**Keywords:** microgravity, *Staphylococcus aureus*, membrane, lipid profiles, antibiotic susceptibility, daptomycin, violacein, SDS

## Abstract

In a survey of the International Space Station (ISS), the most common pathogenic bacterium identified in samples from the air, water and surfaces was *Staphylococcus aureus*. While growth under microgravity is known to cause physiological changes in microbial pathogens, including shifts in antibacterial sensitivity, its impact on *S. aureus* is not well understood. Using high-aspect ratio vessels (HARVs) to generate simulated microgravity (SMG) conditions in the lab, we found *S. aureus* lipid profiles are altered significantly, with a higher presence of branch-chained fatty acids (BCFAs) (14.8% to 35.4%) with a concomitant reduction (41.3% to 31.4%) in straight-chain fatty acids (SCFAs) under SMG. This shift significantly increased the sensitivity of this pathogen to daptomycin, a membrane-acting antibiotic, leading to 12.1-fold better killing under SMG. Comparative assays with two additional compounds, i.e., SDS and violacein, confirmed *S. aureus* is more susceptible to membrane-disrupting agents, with 0.04% SDS and 0.6 mg/L violacein resulting in 22.9- and 12.8-fold better killing in SMG than normal gravity, respectively. As humankind seeks to establish permanent colonies in space, these results demonstrate the increased potency of membrane-active antibacterials to control the presence and spread of *S. aureus*, and potentially other pathogens.

## 1. Introduction

Manned spaceflights function as closed systems, within which a variety of organisms have been identified as originating from humans [1,2]. Microorganisms that thrive within manned spacecraft inhabit surfaces within the life support systems, including water recovery system (WRS), as well as the living environment, impacting astronauts during their exposure [3]. Such long-term and continuous exposure has the potential to develop into serious health threats during future extraterrestrial space missions. These aspects have been continually discussed since the early stages of space exploration, specifically concerning microbial infections among astronauts.

The difficulties in microbial control in microgravity include changes in antimicrobial resistance and the spread of antimicrobial resistance genes. The phenomenon of increased antibiotic resistance due to microgravity has been consistently reported and corroborated by various research groups [4,5]. The spread of antibiotic resistance genes within isolated environments, such as in spacecraft and on the International Space Station (ISS), is an additional concern. Among microorganisms directly isolated from interior spacecraft surfaces, 46 species have been identified, exhibiting resistance to beta-lactam antibiotics, cationic antimicrobial peptides and other antimicrobial agents [6]. In other studies, whole-genome sequencing (WGS) and investigations of antibiotic resistance have been conducted on BSL2 organisms identified on the ISS, resulting in the identification of 123 antimicrobial resistance genes [7]. If present on mobilizable genetic elements, such as conjugative plasmids, antibiotic resistance genes can even be transferred to other bacteria, as was demonstrated with an isolate of *Staphylococcus haemolyticus* from the ISS [4]. The authors noted “(s)peculatively, this transfer could also have happened in multi-species biofilms within the confined ISS/Concordia habitats, thus transferring the *ermC* gene with other traits into more pathogenic bacteria like *S. aureus*”, although they found no evidence that this had occurred in the strains isolated in their study. 

*S. aureus* is of particular concern as a NASA-funded survey of the microbes present in the air, the water or on the surfaces within the ISS from 1998 to 2011 identified many different microbes, but *S. aureus* was the most commonly isolated bacterial species [8]. As an opportunistic pathogen, methicillin-resistant variants of *S. aureus* are listed as a “Serious Threat” by the U.S. Center for Disease Control and Prevention, with an estimated 323,000 infections and 10,600 deaths reported in 2017 in the U.S. alone [9]. This concern is compounded by the finding that bacterial virulence and antibiotic resistance may be enhanced in microgravity conditions [5]. Notably, *Staphylococcus aureus* has demonstrated structural differences under microgravity conditions, including an excessive production of certain components like peptidoglycan, distinguishing it from terrestrial counterparts [10,11]. However, to date, only a few studies have explored how this pathogen responds to microgravity conditions. 

## 2. Materials and Methods

### 2.1. S. aureus Cultivation and Conditioning

Growth of *S. aureus* ATCC 25923 was performed at 30 °C using lysogeny broth (LB) (Difco BD, Detroit, MI, USA). From agar plates, individual colonies were inoculated into LB medium and cultivated overnight in a shaking incubator (250 rpm). These cultures were used as the seeds for the HARVs (RCCS 4H, Synthecon, Houston, TX, USA). 

For the normal gravity (NG) and simulated microgravity (SMG) growth curves, *S. aureus* cultures were grown overnight in flasks initially before being diluted to an OD of 0.03 in 50 mL of sterile LB within the HARVs (RCCS-4H, Synthecon, Houston, TX, USA). After mixing the contents (ten times with a 10 mL syringe) to ensure dispersion of the *S. aureus*, the HARVs were rotated around a vertical or horizontal axis for the NG and SMG conditions, respectively. Each was rotated at a speed of 25 rpm [12,13,14], which generates a low-shear, continuous free-fall environment where the time-averaged gravity vector is reduced to approximately 0.01× *g* [15]. Initially, each culture was pre-conditioned to either NG or SMG by growing them for one day under these same conditions. After growth for 24 h, these pre-conditioned cultures were then used to inoculate fresh HARVs for the experiments. For both cultures, small aliquots (0.5 mL) were taken from the HARVs to determine the ODs and cell viabilities at set times (0, 3, 6, 9, 12 and 24 h) over a 24 h period. 

### 2.2. Fatty Acid Methyl Ester Preparation and Analysis

Analyses of the fatty acids present in the *S. aureus* membrane under both NG and SMG were conducted as described previously [16], albeit with some modification. Briefly, the cultures were grown in the HARVs under NG or SMG for 24 or 48 h. At these times, the cells from each culture (equivalent to 1 mL at OD 3.0) were pelleted (16,000× *g* for 5 min at RT) and washed twice in sterile water. Saponification of the cells was the performed as described previously [17]. For this, 1 mL of Reagent #1 (45 g NaOH (ACS Grade, Sigma-Aldrich, St. Louis, MO, USA), 150 mL HPLC Grade methanol (Sigma-Aldrich, St. Louis, MO, USA) and 150 mL deionized distilled water) was added. After vortexing to suspend the bacteria, this sample was placed at 100 °C for 5 min, vortexed once more and then incubated at 100 °C once more for 25 min. Afterwards, the tubes and samples were cooled down in a room temperature water bath. To initiate methylation of the fatty acids, 2 mL of Reagent #2 (325 mL 6 N HCl (Sigma-Aldrich, St. Louis, MO, USA) mixed with 275 mL HPLC Grade methanol (Sigma-Aldrich, St. Louis, MO, USA) was added to each tube, which was then vortexed and incubated at 80 °C for 10 min. Afterwards, the tubes were quickly cooled by gently shaking them in a room temperature water bath. To each tube, 1.25 mL Reagent #3 (prepared using an equal volume of HPLC Grade hexane (Sigma-Aldrich, St. Louis, MO, USA) and HPLC Grade methyl tert-butyl ether (Sigma-Aldrich, St. Louis, MO, USA) was added and the contents were gently mixed end-over-end for 10 min using a rotator. The aqueous phase was removed from the tubes and discarded. To remove any free fatty acids, the samples were washed with 3 mL of Reagent #4 (10.8 g ACS Grade sodium hydroxide in 900 mL deionized distilled water) by gently mixing them as above for 5 min. Once more, the aqueous phase was removed and discarded. The organic phase was used for gas chromatography (GC) analysis. These samples were then stored at −80 °C until they were analyzed by the Center for Research Facilities at Gyungsang National University.

### 2.3. Chemicals Stocks

Stock solutions of each antimicrobial, i.e., daptomycin, SDS, and violacein, were prepared. SDS was purchased from Sigma-Aldrich (St. Louis, MO, USA) and its stock concentration was 2% (*w*/*v*) in deionized water. After filter sterilization using 0.22 μm Millex^®^ Syringe Filter from Merck Millipore, it was stored at room temperature until needed. Daptomycin was purchased from Thermo Fisher Scientific Inc. (Waltham, MA, USA). Its stock was prepared at a concentration of 4 mg/mL using DMSO (Sigma-Aldrich (St. Louis, MO, USA)) and stored at −20 °C. Violacein extracted from *Chromobacterium violaceum* as described below and was diluted to a concentration of 2.4 mg/mL within DMSO. This stock was then stored at −20 °C until needed. 

### 2.4. Production, Extraction and Quantification of Violacein

Production and purification of violacein was performed as described previously [18,19]. Briefly *Chromobacterium violaceum* ATCC 12742 was cultivated overnight (24 h) in nutrient broth (NB) (Difco BD, Detroit, MI, USA) at 30 °C and 250 rpm in a shaking incubator. To extract the pigment, the bacterial cells were harvested via centrifugation (3600× *g*, 20 min, 4 °C) and violacein was extracted using 95% ethanol (culture volume) by mixing (250 rpm, 6 h, 30 °C). The bacterial cells were then removed by pelleting them (3600× *g*, 10 min, 4 °C). This procedure was repeated until violacein was completely extracted from the cells.

The extract was then filtered (Steritop 45 mm, 0.22 µm PES membrane, Millipore Express PLUS, Millipore, Burlington, MA, USA) to remove any remaining bacterial cells before being dried in a rotary evaporator (N-1110, EYELA, Bohemia, NY, USA) at 50 °C. The dried violacein was the solubilized in 50% acetone and boiled 40 °C within the rotary evaporator until crystallization of the violacein was visually evident. Crystallization was allowed to continue for 24 h at 20 °C. The violacein crystals were collected by centrifugation (7200× *g*, 20 min, 20 °C) and washed with purified water before being dried at 60 °C for 48 h. The purified violacein was then solubilized in DMSO (Sigma-Aldrich) and quantified by HPLC method as described previously [20].

### 2.5. Antimicrobial Tests

For the daptomycin tests in flasks, *S. aureus* was grown overnight as described above. These cultures were then diluted to an optical density (OD_600nm_) of 0.03 in fresh LB media supplemented with both 50 mg/l CaCl_2_ and various concentrations of daptomycin (0, 1, 2 or 4 mg/L). Ca^2+^ was added to the cultures since this ion is known to improve the activities of daptomycin [21,22]. These *S. aureus* cultures were incubated for 9 h at 30 °C and 250 rpm, during which the viability was measured every three hours by plate-counting on LB agar plates.

For the HARV experiments, *S. aureus* was grown under the same conditions to be used (NG or SMG) for 24 h to pre-adapt the culture. Aliquots of these cultures were then inoculated into sterile supplemented LB media (50 mL) in fresh HARVs as described above so the initial OD_600nm_ was 0.03. The LB medium was supplemented with the antibacterial to be tested at the desired concentration before introducing *S. aureus*. Briefly, daptomycin and SDS were diluted using LB medium and introduced into the HARVs (50 mL LB) using a 10 mL sterile syringe. For the tests with daptomycin, CaCl_2_ was also added to a final concentration of 50 mg/L. The medium within the HARVs was then mixed to homogeneity by repeatedly mixing it at least ten times using a 10 mL syringe. For violacein, the stock was diluted in DMSO and 100 µL was added directly to the HARVs. Afterwards, a 10 mL syringe was used to mix the media as above. The final concentrations of DMSO in the HARVs for both daptomycin and violacein was less than 0.2%, a level that had no impact on the viabilities of *S. aureus* [23].

After introducing *S. aureus* into the HARV, the contents were once more mixed at least ten times using a 10 mL syringe. Each culture was then grown for 9 h under the same conditions (NG or SMG) as used for the inoculant. At set times (0, 3, 6 and 9 h), a small aliquot (500 μL) of the culture was sampled using a sterile syringe to determine the viabilities using a plate-counting method. 

### 2.6. Reproducibility and Statistical Analysis

Each experiment was performed in triplicate and the standard deviations are presented on the graphs as error bars. To compare two data groups, statistical analyses were performed using the Student *t*-test. Statistically significant groups at *p*-values of < 0.05, 0.01 or 0.001 are indicated within the figures using asterisks (*, ** or ***, respectively).

## 3. Results and Discussion

### 3.1. Simulated Microgravity Significantly Alters the Membrane Lipid Composition of S. aureus 

**Figure 1:**
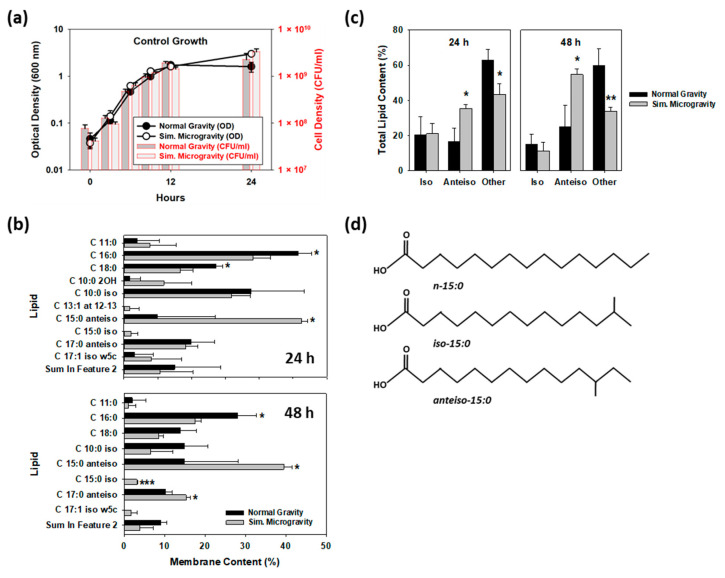
Growth of *S. aureus* under SMG shifts its membrane lipid profiles. (**a**) Growth of *S. aureus* under SMG and NG, showing similar growth rates and cell densities for both. (*n* = 3) (**b**) FAME lipid profiles for the cultures after 24 and 48 h, showing a significant increase in the presence of anteiso-15:0 in the SMG-grown cultures. (*n* = 3) (**c**) These shifts led to a significant increase in the total percentage of anteiso lipids in the *S. aureus* membranes, while that of iso lipids remained unchanged. (*n* = 3) (**d**) Structural representation of the different lipids, showing the presence of a peripheral methyl group within the iso and anteiso lipids. *—*p* < 0.05; **—*p* < 0.01; ***—*p* < 0.001.

Two earlier studies reported that *S. aureus* grew equally well in both simulated microgravity (SMG) and normal gravity (NG) conditions [24,25]. This was also true here in our study, as shown in Figure 1a, where both the measured optical densities and cell densities (colony-forming units (CFU)/mL) are plotted. While no differences in *S. aureus* growth rates were evident, SMG is known to induce other physiological changes. For instance, SMG growth altered the membrane lipid composition in vastly different organisms, including *E. coli* [26], *Listeria monocytogenes* [27] and even plants, such as *Pisum sativum* (snow peas) [28]. Similarly, although the lipid composition was not determined, proteome analyses of the black fungus *Knufia chersonesos* found genes involved in unsaturated fatty acid biosynthesis and the metabolism of phospho- and glycerophospholipids were upregulated under SMG conditions [29], suggesting it also experiences shifts in its lipid/fatty acid contents. 

Extending those findings to our study, we hypothesized the fatty acid composition in *S. aureus*’ membranes may experience a similar shift under NG and SMG conditions and, consequently, they were analyzed. Figure 1b shows the fatty acids present in the NG and SMG cultures clearly differed at both 24 and 48 h. The predominant saturated straight chain fatty acids (SCFAs) found in the 24-h NG cultures were C16:0 (25.8%) and C18:0 (13.6%), while iso-C10:0 (18.8%) and anteiso-C17:0 (9.9%) were the most common branched chain fatty acids (BCFAs). Under SMG, however, the percentage of C16:0 and C18:0 dropped significantly (to 19.1% and 8.4%, respectively), while that of anteiso-C15:0 increased 530% (from 4.9% in NG to 26.3% under SMG), making it the most common fatty acid within *S. aureus* under these conditions. Similar results were obtained with the 48-h cultures except a second BCFA, i.e., anteiso-C17:0, was also significantly higher in the SMG culture.

When the total fatty acid composition was considered, the population of iso-BCFAs in both NG and SMG cultures were identical for both time points, but that of the anteiso-BCFAs were always significantly higher in the SMG cultures (Figure 1c). These results parallel those found previously with *L. monocytogenes* [27], where anteiso-BCFA percentages in the membrane increased under SMG, leading to greater membrane fluidity in this pathogen. In their study with *L. monocytogenes*, the iso/anteiso- branched chain ratio decreased from 0.448 under NG to 0.392 in SMG, leading to greater membrane fluidity [27]. Similarly, with *S. aureus*, we found similar results as the iso/anteiso- branched chain ratio decreased from 1.24 under NG to 0.60 under SMG. Moreover, the significant drop in C16:0 and C18:0 abundance seen here under SMG, where these two fatty acids drop by nearly 12% (i.e., from 39.4 ± 3.0% under normal gravity to 27.5 ± 4.3% in SMG (*p* = 0.017)), also increases membrane fluidity. As described in the study by Mostofian, et al. [30], the loss of these SCFAs and their replacement by BCFAs act to increase the in-plane mean-square displacement of individual lipids, i.e., each lipid within the membrane is more mobile and moves larger average distances in a given amount of time. All of these results indicate SMG growth of *S. aureus* leads to increased membrane fluidity, an attribute that can be exploited to control this pathogen within microgravity environments.

### 3.2. Daptomycin Is More Active against S. aureus under SMG Conditions

A study by Rosado, et al. [31] reported SMG growth does not impact the sensitivity of *S. aureus* to several different antibiotics, i.e., erythromycin (a protein synthesis inhibitor), or two cell wall synthesis inhibitors (flucloxacillin and vancomycin). However, the results shown in Figure 1c led us to hypothesize that this would not necessarily hold true for daptomycin. 

Daptomycin is a lipopeptide antibiotic that integrates into the cellular membrane, where it aggregates and causes membrane disruption and leaking, eventually leading to cell death [22,32]. Although the exact mechanism by which daptomycin kills Gram-positive bacterial pathogens is not fully understood, it is known that daptomycin preferentially interacts with disordered regions within the membrane, referred to regions of increased fluidity in one study [33]. This phenomenon was also demonstrated using several different strains of *S. aureus*, where differences in their membrane fatty acid composition were found to directly determine the efficacy of daptomycin against each strain [34]. In their study, they found the fatty acid percentages and the susceptibility of the strains correlated, with susceptible strains possessing large percentages of anteiso fatty acids within their membranes than strains that were resistant. Conversely, strains that had a higher percentage of SCFAs in their membranes, which leads to a more structured/rigid membrane [30], were more resistant to daptomycin [34]. 

**Figure 2:**
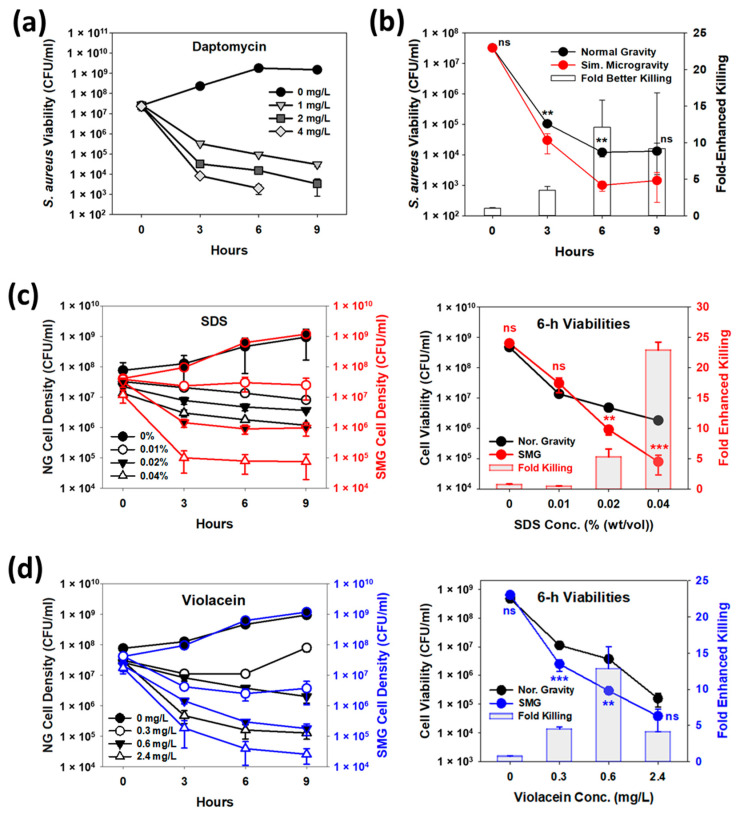
Increased potency of membrane-disrupting agents under SMG conditions. (**a**) Daptomycin dose-dependency against *S. aureus* cultures grown in flasks. Based on these results, a concentration of 2 mg/L daptomycin was selected for NG and SMG tests. (*n* = 3) (**b**) Daptomycin activity against *S. aureus* significantly increases under SMG. The results show six hours gave the greatest improvement in bactericidal activities. (*n* = 3) (**c**) SDS was also more potent under SMG, with 0.02% and 0.04% leading to significantly better killing of *S. aureus* (5.1- to 22.9-fold enhanced killing, respectively). (*n* = 3) (**d**) Violacein activities against *S. aureus* were also significantly better, with 0.6 mg/L violacein showing 12.8-fold enhanced killing of *S. aureus*. ns—not significant; **—*p* < 0.01; ***—*p* < 0.001.

Extending these findings to our study, the significantly higher percentage of anteiso fatty acids in *S. aureus* membrane under SMG conditions (Figure 1c) should likewise increase the sensitivities of these cultures to daptomycin. To explore this, we first determined what concentration of daptomycin was effective against our *S. aureus* strain. As shown in Figure 2a, daptomycin killing was dose-dependent in flask tests. While 4 mg/L led to better killing than 2 mg/L, the viability of *S. aureus* after 9 h was below 100 CFU/mL, which was considered too low to explore improved activities and, consequently, 2 mg/L was chosen for the HARV studies. 

In these experiments, we found *S. aureus* grown under SMG conditions was significantly more susceptible to daptomycin (Figure 2b), results that agree with the those noted above [33,34], namely that the greater presence of anteiso fatty acids increases daptomycin’s binding and disruption of the membrane. The greatest difference in activity was seen at 6 h, where daptomycin killing was 12.1-fold better against SMG-cultures of *S. aureus* and, thus, this time was used in later experiments.

### 3.3. Increased Potency of SDS and Violacein against SMG-Grown S. aureus

While our results above clearly show daptomycin activities are better against SMG-grown *S. aureus*, suggesting it may also be more potent in microgravity environments, the use of this antibiotic faces some hurdles. One concern, for instance, is the shorter lifespan of pharmaceuticals within low earth orbit (LEO), such as on the ISS. Based on the study by Du, et al. [35], pharmaceuticals tend to lose their potency faster within space, the authors attributing this to greater high energy radiation exposures. More recently, Reichard, et al. [36] analyzed this and several other drug stability studies and concluded the probability for failure of active pharmaceutical ingredients is greater in LEO, but also suggested that suitable protective repackaging strategies can help alleviate this problem. A second, and potentially greater, concern is that daptomycin is regarded as a last-resort option to treat multidrug-resistant pathogens and their infections [37]. As such, to reduce the chances for daptomycin resistance to spread, its use should be limited to only those cases where other options are not available. As noted above, *S. aureus* was the most common bacterial strain found within environmental samples taken from the ISS [8]. As such, rather than focusing on treating infections once they occur, it might be better to take a prophylactic approach to control this pathogen within microgravity environments. Towards this end, we explored if other membrane-disrupting antibacterials also see an increase in their potency. 

The first option considered was SDS, a surfactant with strong bactericidal activities against Gram-positive bacteria [38,39], including *S. aureus*, for which the minimum inhibitory concentration (MIC) is 0.01% (*w*/*v*) [40]. Much like daptomycin, SDS is known to insert into and disrupt the membrane and its integrity. While the impact of SDS on more fluid membranes has not been reported previously, lipids in fluid membranes have greater lateral mobility [30], allowing SDS molecules to easily incorporate into the lipid bilayer. We hypothesized, therefore, that reduced lipid packing in fluid membranes provides more opportunities for SDS molecules to insert into, interact with and disrupt the lipid bilayer structure. Based on the FAME analyses in Figure 1, this should extend to *S. aureus* under SMG conditions. Using a range of SDS concentrations (0~0.04%), we found this to be the case as dose-dependent bactericidal activities were seen with SDS in both NG and SMG, but significantly better killing occurred in SMG cultures (Figure 2c). This was especially true for the higher concentrations tested, i.e., 0.02% and 0.04%, which led to 5.3- and 22.9-fold better killing at 6 h under SMG conditions than NG.

The second membrane-disrupting compound evaluated was violacein, a hydrophobic bisindole molecule produced by a range of bacterial strains [41]. While the biological activities of violacein are diverse [42], including as an anticancer [43,44] and antiviral [45], it is probably best known for its antibacterial activities against Gram-positive pathogens [17,20,42,46,47]. This activity is due to its hydrophobic nature. Vioacein’s reported octanol: water partitioning coefficient (LogP_octanol:water_ value) is 3.34 and, as such, it preferably and rapidly inserts into lipid membranes, where it causes massive disruption of *S. aureus*’ membrane integrity, leading to a leakage of ions and proteins [48,49].

As with SDS, we hypothesized that the more disordered membrane state in SMG-grown *S. aureus* cells would increase the potency of violacein against this pathogen. As shown in Figure 2d, the lower concentrations of violacein tested (0.3 and 0.6 mg/L) were significantly better at killing *S. aureus* under SMG, i.e., the *S. aureus* viabilities dropped by factors of 4.5 and 12.8 when compared against NG, respectively. In contrast, while better killing was also seen with 2.4 mg/L violacein (4.1-fold enhanced killing at 6 h), the results were not significantly different from tests performed under NG. This finding suggests the benefits offered by shifts in the membrane fatty acid composition diminish and violacein’s killing activities tend to consolidate as more of this compound is applied.

### 3.4. Potential for Co-Treatment

While the data presented above show positive results for daptomycin, SDS and violacein, the activity spectrums for all three antibacterials are primarily against Gram-positive bacterial strains since, owing to their outer membranes, Gram-negative strains and pathogens tend to be much more resistant [41,42,50,51,52,53]. As surveys found many different Gram-negative pathogens on the ISS as well [6,7,8], one possible avenue to expand the current findings is to consider a co-application of these antibacterials with others that also target Gram-negative bacterial species. One example of this is the study Im, et al. [46], where violacein was successfully used alongside the predatory bacterium, *Bdellovibrio bacteriovorus*, against polymicrobial populations, leading to better killing when both were used together. While that study did not explore SMG conditions, a separate study by Jang, et al. [54] found *B. bacteriovorus* is not only capable of predating in SMG environments, it was more active for some of the pathogens tested (carbapenem-resistant strains *Klebsiella pneumoniae* and *Acinetobacter baumannii*), i.e., approximately 1-log better killing under SMG than NG. Predation also provided a hidden benefit as *B. bacteriovorus* significantly reduced (by more than 97.8%) the antibiotic resistance genes present in these pathogens (*bla*_KPC-2_ and *bla*_OXA-51_, respectively). While these results are impressive and encouraging, *B. bacteriovorus* represents only one option that was considered so far, with many more possible.

## 4. Conclusions

Understanding the physiological changes microgravity imparts on pathogenic microorganisms, and the subsequent impact and alteration these have on antimicrobial efficacies, is clearly vital in maintaining astronaut health during long-duration space missions. Within this study, we found growth of *S. aureus* under SMG led to significant changes in its membrane fatty acid profiles, with a strong increase in the percentage of anteiso fatty acids. As higher percentages of anteiso fatty acids are known to increase membrane disorder and fluidity [33,34], we exploited this physiological shift to demonstrate three distinct membrane-disrupting antibacterials (i.e., daptomycin, SDS and violacein) are all more potent against SMG-cultured *S. aureus*. While the results presented here suggest the use of daptomycin in the treatments of Gram-positive pathogens and their infections may be more successful within microgravity environments, and also that prophylactic measures, such as the use of detergents to clean surfaces, may be more effective, this study does present some limitations. One clear limitation in the present study is these experiments were not performed in LEO, but within HARVs. Validation of these results within true microgravity conditions, therefore, is needed. In addition, the sub-cellular mechanisms governing the lipid profile shifts should be explored, potentially through gene expression analyses.

## Data Availability

Any additional data will be provided upon request.

## References

[1] Checinska Sielaff A., Urbaniak C., Mohan G.B.M., Stepanov V.G., Tran Q., Wood J.M., Minich J., McDonald D., Mayer T., Knight R. (2019). Characterization of the total and viable bacterial and fungal communities associated with the International Space Station surfaces. Microbiome.

[2] Mora M., Wink L., Kögler I., Mahnert A., Rettberg P., Schwendner P., Demets R., Cockell C., Alekhova T., Klingl A. (2019). Space Station conditions are selective but do not alter microbial characteristics relevant to human health. Nat. Commun..

[3] Zea L., McLean R.J.C., Rook T.A., Angle G., Carter D.L., Delegard A., Denvir A., Gerlach R., Gorti S., McIlwaine D. (2020). Potential biofilm control strategies for extended spaceflight missions. Biofilm.

[4] Schiwon K., Arends K., Rogowski K.M., Fürch S., Prescha K., Sakinc T., Van Houdt R., Werner G., Grohmann E. (2013). Comparison of Antibiotic Resistance, Biofilm Formation and Conjugative Transfer of Staphylococcus and Enterococcus Isolates from International Space Station and Antarctic Research Station Concordia. Microb. Ecol..

[5] Nickerson C.A., O’Brien A.D., Ott C.M., Mister S.J., Morrow B.J., Burns-Keliher L., Pierson D.L. (2000). Microgravity as a Novel Environmental Signal Affecting Salmonella enterica Serovar Typhimurium Virulence. Infect. Immun..

[6] Singh N.K., Wood J.M., Karouia F., Venkateswaran K. (2018). Succession and persistence of microbial communities and antimicrobial resistance genes associated with International Space Station environmental surfaces. Microbiome.

[7] Urbaniak C., Sielaff A.C., Frey K.G., Allen J.E., Singh N., Jaing C., Wheeler K., Venkateswaran K. (2018). Detection of antimicrobial resistance genes associated with the International Space Station environmental surfaces. Sci. Rep..

[8] Pierson D.L., Botkin D.J., Bruce R.J., Castro V.A., Smith M.J., Oubre C.M., Ott C.M. Microbial monitoring of the international space station. Proceedings of the 8th International Workshop on Space Microbiology (No. JSC-CN-28760).

[9] CDC U.S. (2019). Antibiotic Resistance Threats in the United States. www.cdc.gov/drugresistance/pdf/threats-report/2019-ar-threats-report-508.pdf.

[10] Tixador R., Richoilley G., Gasset G., Planel H., Moatti N., Lapchine L., Enjalbert L., Raffin J., Bost R., Zaloguev S.N. (1985). Preliminary results of cytos 2 experiment. Acta Astronaut..

[11] Lapchine L., Moatti N., Gasset G., Richoilley G., Templier J., Tixador R. (1986). Antibiotic activity in space. Drugs Exp. Clin. Res..

[12] Kim H.W., Matin A., Rhee M.S. (2014). Microgravity Alters the Physiological Characteristics of *Escherichia coli* O157:H7 ATCC 35150, ATCC 43889, and ATCC 43895 under Different Nutrient Conditions. Appl. Environ. Microbiol..

[13] Wilson J.W., Ramamurthy R., Porwollik S., McClelland M., Hammond T., Allen P., Ott C.M., Pierson D.L., Nickerson C.A. (2002). Microarray analysis identifies Salmonella genes belonging to the low-shear modeled microgravity regulon. Proc. Natl. Acad. Sci. USA.

[14] Tirumalai M.R., Karouia F., Tran Q., Stepanov V.G., Bruce R.J., Ott C.M., Pierson D.L., Fox G.E. (2017). The adaptation of *Escherichia coli* cells grown in simulated microgravity for an extended period is both phenotypic and genomic. npj Microgravity.

[15] Unsworth B.R., Lelkes P.I. (1998). Growing tissues in microgravity. Nat. Med..

[16] Sasser M. (1990). Identification of Bacteria by Gas Chromatography of Cellular Fatty Acids.

[17] Choi S.Y., Kim S., Lyuck S., Kim S.B., Mitchell R.J. (2015). High-level production of violacein by the newly isolated *Duganella violaceinigra* str. NI28 and its impact on *Staphylococcus aureus*. Sci. Rep..

[18] Rodrigues A.L., Göcke Y., Bolten C., Brock N.L., Dickschat J.S., Wittmann C. (2011). Microbial production of the drugs violacein and deoxyviolacein: Analytical development and strain comparison. Biotechnol. Lett..

[19] Rodrigues A.L., Trachtmann N., Becker J., Lohanatha A.F., Blotenberg J., Bolten C.J., Korneli C., de Souza Lima A.O., Porto L.M., Sprenger G.A. (2013). Systems metabolic engineering of *Escherichia coli* for production of the antitumor drugs violacein and deoxyviolacein. Metab. Eng..

[20] Choi S.Y., Lim S., Cho G., Kwon J., Mun W., Im H., Mitchell R.J. (2020). *Chromobacterium violaceum* delivers violacein, a hydrophobic antibiotic, to other microbes in membrane vesicles. Environ. Microbiol..

[21] LaPlante K.L., Rybak M.J. (2005). Daptomycin—A novel antibiotic against Gram-positive pathogens. Expert Opin. Pharmacother..

[22] Jung D., Rozek A., Okon M., Hancock R.E.W. (2004). Structural transitions as determinants of the action of the calcium-dependent antibiotic daptomycin. Chem. Biol..

[23] Zgoda J.R., Porter J.R. (2008). A Convenient Microdilution Method for Screening Natural Products Against Bacteria and Fungi. Pharm. Biol..

[24] Hauserman M.R., Rice K.C. (2021). Growth of *Staphylococcus aureus* Using a Rotary Cell Culture System. Staphylococcus aureus: Methods in Molecular Biology.

[25] Singh S., Vidyasagar P.B., Kulkarni G.R. (2021). Investigating alterations in the cellular envelope of *Staphylococcus aureus* in simulated microgravity using a random positioning machine. Life Sci. Space Res..

[26] Kim H.W., Rhee M.S., Kelly R.M. (2016). Influence of Low-Shear Modeled Microgravity on Heat Resistance, Membrane Fatty Acid Composition, and Heat Stress-Related Gene Expression in *Escherichia coli* O157:H7 ATCC 35150, ATCC 43889, ATCC 43890, and ATCC 43895. Appl. Environ. Microbiol..

[27] Sheet S., Yesupatham S., Ghosh K., Choi M.-S., Shim K.S., Lee Y.S. (2020). Modulatory effect of low-shear modeled microgravity on stress resistance, membrane lipid composition, virulence, and relevant gene expression in the food-borne pathogen Listeria monocytogenes. Enzym. Microb. Technol..

[28] Nedukha O., Kordyum E., Vorobyova T. (2021). Sensitivity of Plant Plasma Membrane to Simulated Microgravity. Microgravity Sci. Technol..

[29] Tesei D., Chiang A.J., Kalkum M., Stajich J.E., Mohan G.B.M., Sterflinger K., Venkateswaran K. (2021). Effects of Simulated Microgravity on the Proteome and Secretome of the Polyextremotolerant Black Fungus *Knufia chersonesos*. Front. Genet..

[30] Mostofian B., Zhuang T., Cheng X., Nickels J.D. (2019). Branched-Chain Fatty Acid Content Modulates Structure, Fluidity, and Phase in Model Microbial Cell Membranes. J. Phys. Chem. B.

[31] Rosado H., Doyle M., Hinds J., Taylor P.W. (2010). Low-shear modelled microgravity alters expression of virulence determinants of *Staphylococcus aureus*. Acta Astronaut..

[32] Straus S.K., Hancock R.E.W. (2006). Mode of action of the new antibiotic for Gram-positive pathogens daptomycin: Comparison with cationic antimicrobial peptides and lipopeptides. Biochim. Biophys. Acta (BBA) Biomembr..

[33] Müller A., Wenzel M., Strahl H., Grein F., Saaki T.N.V., Kohl B., Siersma T., Bandow J.E., Sahl H.-G., Schneider T. (2016). Daptomycin inhibits cell envelope synthesis by interfering with fluid membrane microdomains. Proc. Natl. Acad. Sci. USA.

[34] Boudjemaa R., Cabriel C., Dubois-Brissonnet F., Bourg N., Dupuis G., Gruss A., Lévêque-Fort S., Briandet R., Fontaine-Aupart M.-P., Steenkeste K. (2018). Impact of Bacterial Membrane Fatty Acid Composition on the Failure of Daptomycin to Kill *Staphylococcus aureus*. Antimicrob. Agents Chemother..

[35] Du B., Daniels V.R., Vaksman Z., Boyd J.L., Crady C., Putcha L. (2011). Evaluation of Physical and Chemical Changes in Pharmaceuticals Flown on Space Missions. AAPS J..

[36] Reichard J.F., Phelps S.E., Lehnhardt K.R., Young M., Easter B.D. (2023). The effect of long-term spaceflight on drug potency and the risk of medication failure. npj Microgravity.

[37] Steenbergen J.N., Alder J., Thorne G.M., Tally F.P. (2005). Daptomycin: A lipopeptide antibiotic for the treatment of serious Gram-positive infections. J. Antimicrob. Chemother..

[38] Shen Y., Li P., Chen X., Zou Y., Li H., Yuan G., Hu H. (2020). Activity of Sodium Lauryl Sulfate, Rhamnolipids, and N-Acetylcysteine Against Biofilms of Five Common Pathogens. Microb. Drug Resist..

[39] Díaz De Rienzo M.A., Stevenson P., Marchant R., Banat I.M., Fanning S. (2016). Antibacterial properties of biosurfactants against selected Gram-positive and -negative bacteria. FEMS Microbiol. Lett..

[40] Msadek T., Makgotlho P.E., Marincola G., Schäfer D., Liu Q., Bae T., Geiger T., Wasserman E., Wolz C., Ziebuhr W. (2013). SDS Interferes with SaeS Signaling of *Staphylococcus aureus* Independently of SaePQ. PLoS ONE.

[41] Choi S.Y., Yoon K.-h., Lee J.I., Mitchell R.J. (2015). Violacein: Properties and Production of a Versatile Bacterial Pigment. BioMed Res. Int..

[42] Choi S.Y., Lim S., Yoon K.-h., Lee J.I., Mitchell R.J. (2021). Biotechnological Activities and Applications of Bacterial Pigments Violacein and Prodigiosin. J. Biol. Eng..

[43] Liekens S., Schols D., Hatse S. (2010). CXCL12-CXCR4 axis in angiogenesis, metastasis and stem cell mobilization. Curr. Pharm. Des..

[44] Platt D., Amara S., Mehta T., Vercuyssee K., Myles E.L., Johnson T., Tiriveedhi V. (2014). Violacein inhibits matrix metalloproteinase mediated CXCR4 expression: Potential anti-tumor effect in cancer invasion and metastasis. Biochem. Biophys. Res. Commun..

[45] Andrighetti-Frohner C.R., Antonio R.V., Creczynski-Pasa T.B., Barardi C.R., Simoes C.M. (2003). Cytotoxicity and potential antiviral evaluation of violacein produced by *Chromobacterium violaceum*. Memórias Inst. Oswaldo Cruz.

[46] Im H., Choi S.Y., Son S., Mitchell R.J. (2017). Combined Application of Bacterial Predation and Violacein to Kill Polymicrobial Pathogenic Communities. Sci. Rep..

[47] Nakamura Y., Asada C., Sawada T. (2003). Production of antibacterial violet pigment by psychrotropic bacterium RT102 strain. Biotechnol. Bioprocess Eng..

[48] Aruldass C.A., Masalamany S.R.L., Venil C.K., Ahmad W.A. (2018). Antibacterial mode of action of violacein from *Chromobacterium violaceum* UTM5 against *Staphylococcus aureus* and methicillin-resistant *Staphylococcus aureus* (MRSA). Environ. Sci. Pollut. Res..

[49] Cauz A.C.G., Carretero G.P.B., Saraiva G.K.V., Park P., Mortara L., Cuccovia I.M., Brocchi M., Gueiros-Filho F.J. (2019). Violacein Targets the Cytoplasmic Membrane of Bacteria. ACS Infect. Dis..

[50] Cho G., Kwon J., Soh S.M., Jang H., Mitchell R.J. (2019). Sensitivity of predatory bacteria to different surfactants and their application to check bacterial predation. Appl. Microbiol. Biotechnol..

[51] Tally F.P., Zeckel M., Wasilewski M.M., Carini C., Berman C.L., Drusano G.L., Oleson F.B. (2005). Daptomycin: A novel agent for Gram-positive infections. Expert Opin. Investig. Drugs.

[52] Lahiri A., Ananthalakshmi T.K., Nagarajan A.G., Ray S., Chakravortty D. (2011). TolA mediates the differential detergent resistance pattern between the *Salmonella enterica* subsp. enterica serovars Typhi and Typhimurium. Microbiology.

[53] Kramer V.C., Nickerson K.W., Hamlett N.V., O’Hara C. (1984). Prevalence of extreme detergent resistance among the Enterobacteriaceae. Can. J. Microbiol..

[54] Jang H., Choi S.Y., Mun W., Jeong S.H., Mitchell R.J. (2022). Predation of colistin- and carbapenem-resistant bacterial pathogenic populations and their antibiotic resistance genes in simulated microgravity. Microbiol. Res..

